# Revision Anterior Cruciate Ligament Reconstruction in an Adult Twin With Meier-Gorlin Syndrome

**DOI:** 10.31486/toj.25.0048

**Published:** 2026

**Authors:** Misty Suri, Srikanth Mudiganty, Salvatore Sclafani, David Vanegas-Contla, Brian Godshaw, Scott Montgomery

**Affiliations:** ^1^ Department of Orthopedics and Sports Medicine, Ochsner Clinic Foundation, New Orleans, LA; ^2^ Department of Orthopedics, Hospital Excel, Tijuana, Baja California, Mexico; ^3^ The University of Queensland Medical School, Ochsner Clinical School, New Orleans, LA

**Keywords:** *Absent patella*, *anterior cruciate ligament reconstruction*, *joint instability*, *Meier-Gorlin syndrome*, *reoperation*

## Abstract

**Background:**

Meier-Gorlin syndrome is a rare autosomal recessive disorder characterized by the clinical triad of microtia, patellar aplasia or hypoplasia, and proportionate short stature. The absence of the patella poses 2 surgical challenges: lack of a donor site for graft harvest and lack of anterior restraint during anterior cruciate ligament (ACL) reconstruction surgery. Given that individuals with Meier-Gorlin syndrome generally exhibit normal cognitive function and life expectancy, ensuring knee stability is essential to support their ability to participate in sports and engage in daily activities independently.

**Case Report:**

We present the case of an adult twin with Meier-Gorlin syndrome who had an absent patella and recurrent knee instability after ACL retear. At 2-year follow-up after revision ACL reconstruction, the patient's Single Assessment Numeric Evaluation score had increased to 100 from a preoperative score of 30. The patient reported excellent pain relief and was able to perform all activities of daily living.

**Conclusion:**

Successful revision ACL reconstruction is possible in a patient with Meier-Gorlin syndrome. However, these patients may be at higher risk of revision because of the lack of the anterior constraint provided by a normal extensor mechanism with a present patella.

## INTRODUCTION

The anterior cruciate ligament (ACL) is the most injured ligament in the knee. ACL tears account for half of all knee injuries, with an estimated annual incidence in the United States of 1 in 3,500 individuals. Approximately 400,000 ACL reconstructions are performed in the United States each year.^1,2^ Risk factors for ACL injury include female sex, increased body mass index, impingement at the intercondylar notch, hypermobility, and joint laxity.^1^ Compared to males, female athletes are more at risk of ACL injury because they tend to have a smaller ACL and smaller intercondylar notch, to have weaker core stability, to have increased valgus angulation and extension of the knee with landing mechanics, and to be more quadriceps dominant.^1^ Other risk factors for ACL injury may include higher posterolateral tibial slope and higher baseline activity level.^1,3^

Short- and long-term follow-up studies have demonstrated satisfactory results in patients who undergo ACL reconstruction.^4,5^ Wright et al reported an equal risk of 3% for ACL graft rupture and contralateral normal knee ACL rupture at 2-year follow-up.^6^ Sanders et al reported a 9% rate of graft failure after ACL reconstruction in their 25-year follow-up study.^7^ The incidence of revision after ACL reconstruction is estimated to be 7.7%.^8^

Risk factors for ACL graft failures are not as well understood as those for native ACL injury. However, use of allograft for reconstruction, smaller graft diameter, previous ACL surgery, return to high activity level or sport, poor surgical technique, undiagnosed concurrent knee injuries, and failed biologic incorporation of the graft have been found to contribute to ACL graft failure.^1,9,10^

Meier-Gorlin syndrome or ear-patella-short stature syndrome is a rare autosomal recessive disorder characterized by the clinical triad of microtia, patellar aplasia or hypoplasia, and proportionate short stature. At least 2 of these clinical features are present in 97% of patients with Meier-Gorlin syndrome, with a small or absent patella being the most prevalent manifestation. Other clinical features include a small mouth with full lips, microretrognathia (a small, backward positioned lower jaw), and microcephaly. These manifestations may be accompanied by respiratory, urogenital, and cardiac anomalies. Intellect and life expectancy are typically normal for most individuals.^11^

The exact prevalence of Meier-Gorlin syndrome has not been determined but is estimated to be less than 1 to 9 per 1,000,000.^11^ Mutations in 1 of 5 genes (ORC1, ORC4, ORC6, CDT1, and CDC6) involved in DNA replication are found in approximately 67% to 78% of patients with this disorder.^11^ Patients with mutations of the ORC1 and ORC4 genes have the most severe short stature and microcephaly. In a patient suspected of having Meier-Gorlin syndrome, a confirmatory diagnosis can be obtained by detecting mutations in 1 of the 5 prereplication complex genes (ORC1, ORC4, ORC6, CDT1, and CDC6).^11^

Because Meier-Gorlin syndrome is a rare disorder, literature on the natural history of associated orthopedic manifestations is scarce. Literature on the biomechanical consequences of an absent patella on ligament stability and tibiofemoral joint stress is limited. Although the loss of the patella through patellectomy is different from congenital patellar aplasia/hypoplasia, patellectomy has been shown to alter knee joint biomechanics, increasing tibiofemoral stresses and contributing to ligamentous instability and degenerative changes.^12,13^ Sutton et al evaluated the effects of patellectomy on knee function in patients who underwent treatment for traumatic fracture of the patella, patellofemoral arthritis, or chondromalacia of the patella. The authors found clinical instability in 4 of 11 knees with partial patellectomy and 19 of 26 knees with complete patellectomy, with a greater degree of instability occurring in knees with complete patellectomy.^12^ In their systematic review, Cavaignac et al reported tibiofemoral arthritis to be the second most common complication reported after patellectomy.^13^

A patient with Meier-Gorlin syndrome can present challenges to a successful surgical outcome. Because patients with Meier-Gorlin syndrome typically have normal life expectancies, the goal of operative management is to provide a functional and stable knee for the long term. Because of the aplasia or hypoplasia of the patella and what may be considered an already-compromised extensor mechanism, ACL reconstruction using hamstring autograft or allograft may be preferred to quadriceps autograft.

To our knowledge, no cases of ACL reconstruction in a patient with Meier-Gorlin syndrome or an absent patella have been reported. We present the case of a revision ACL reconstruction in a patient with Meier-Gorlin syndrome and an absent patella with a 2-year clinical follow-up.

## CASE REPORT

A 30-year-old female with Meier-Gorlin syndrome injured her left knee while cheerleading in high school in 2011 and underwent arthroscopic meniscus repair surgery at an outside facility. Six months later, because of instability, she underwent successful arthroscopic ACL reconstruction with a posterior tibial tendon allograft of the left knee. One year later, she experienced pain and instability in the same knee, possibly following another episode of trauma, and underwent repeat knee arthroscopy with partial meniscectomy and imbrication of the ACL.

**Figure 1.**
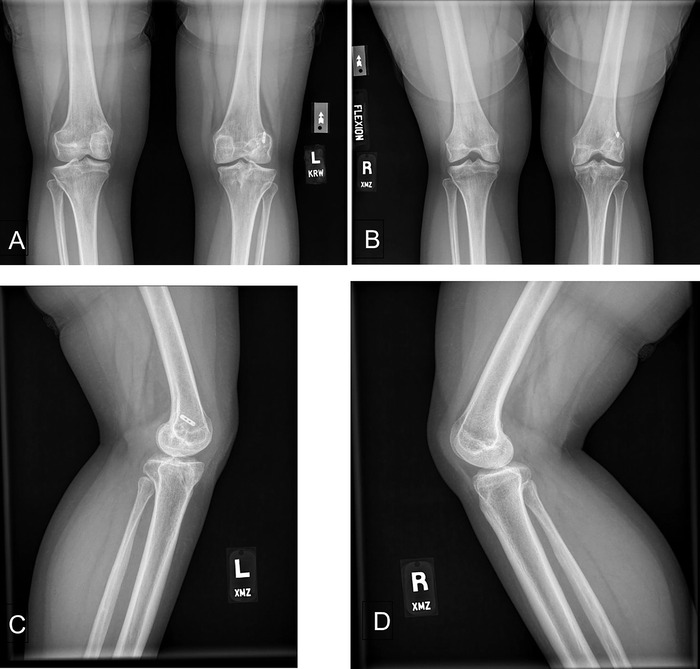
Preoperative anteroposterior radiographs of both knees in (A) standing view and (B) at 45° flexion show prior anterior cruciate ligament reconstruction tunnels along with Endobutton fixation device (Smith & Nephew plc) over the lateral distal femur. Lateral radiographs of the (C) left and (D) right knees show patellar aplasia.

**Figure 2.**
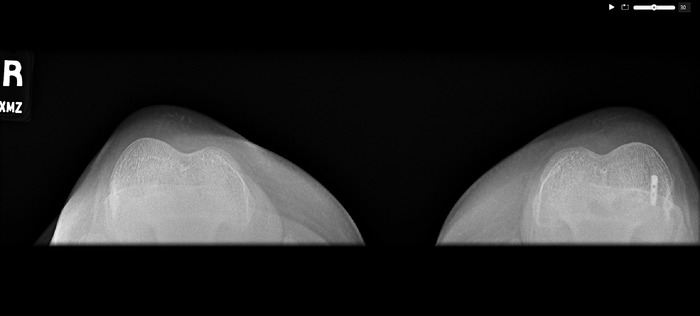
Skyline radiograph of both knees shows patellar aplasia.

**Figure 3.**
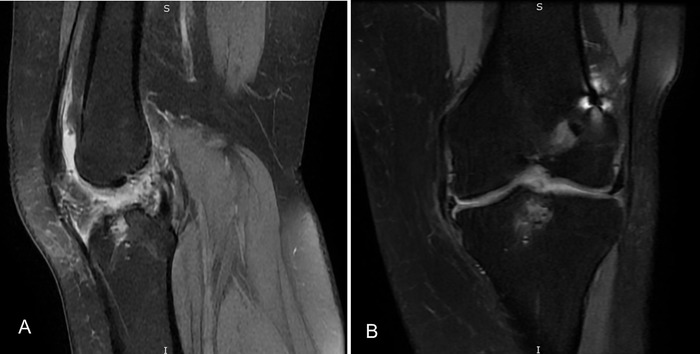
Magnetic resonance imaging of the left knee in (A) sagittal view shows complete tear of the anterior cruciate ligament (ACL) and (B) coronal view shows femur and tibia tunnels of previous ACL reconstruction with complete ACL tear and an absent patella.

**Figure 4.**
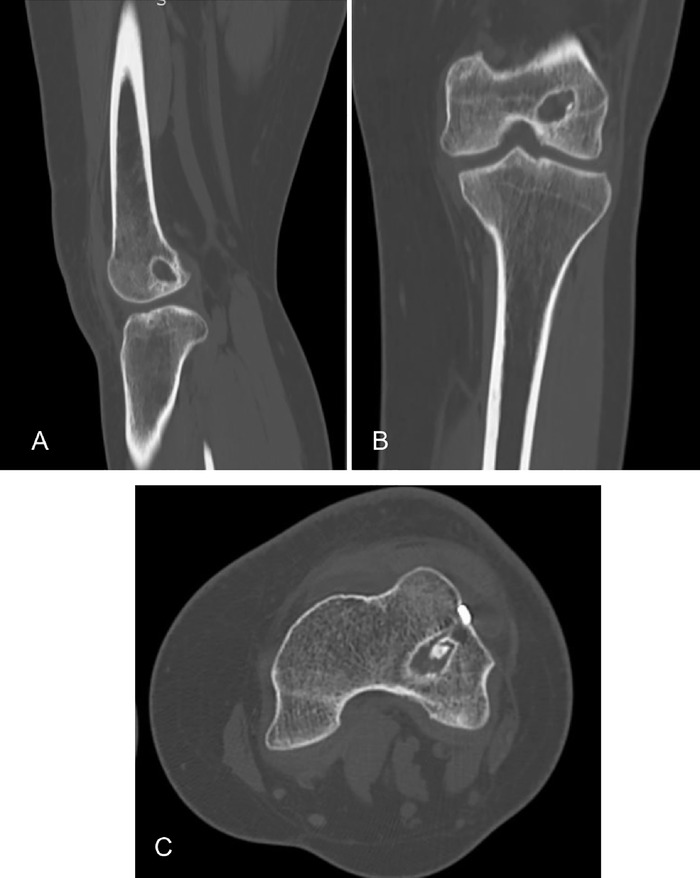
Computed tomography scans of the left knee show femur tunnel in (A) sagittal, (B) coronal, and (C) axial views.

**Figure 5.**
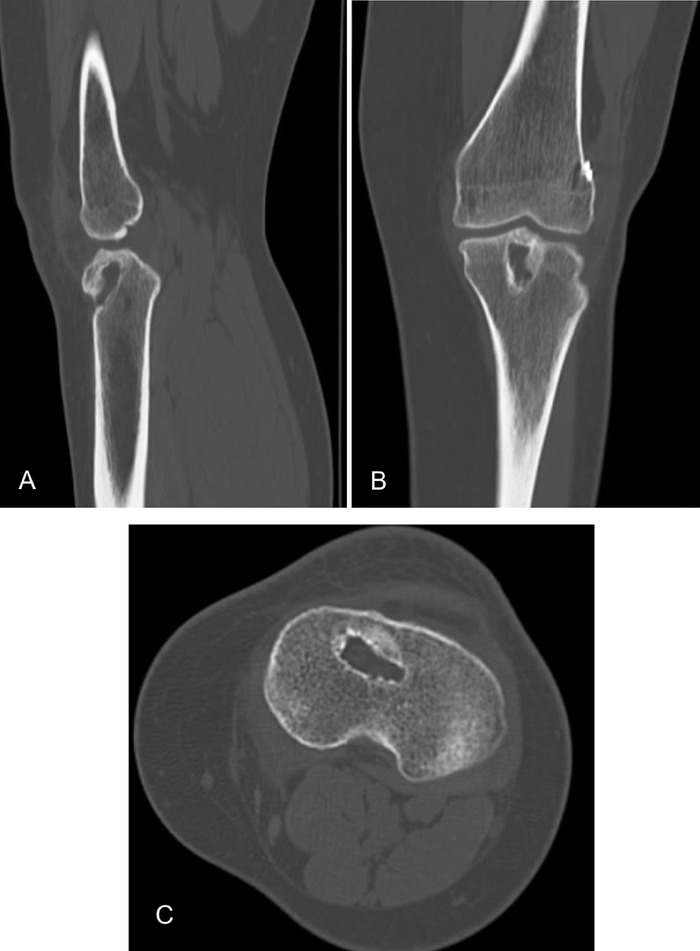
Computed tomography scans of the left knee show tibia tunnel in (A) sagittal, (B) coronal, and (C) axial views.

At the time of presentation to our clinic, she complained of left knee pain, swelling, and instability for 3 weeks with a history of antecedent trauma. While walking on uneven ground in the park, the patient sustained a twisting injury to her knee. Physical examination of the left knee revealed tenderness along the medial joint line. Range of motion was comparable to the opposite side with flexion of 145° and hyperextension of 5°. Pain was noted on terminal flexion. McMurray test was positive, and Lachman test revealed grade 2B laxity. Radiographs of the knee showed signs of previous ACL reconstruction and absence of the patella (Figures 1 and 2). The Endobutton fixation device (Smith & Nephew plc) was visualized along the cortex of the distal femoral lateral metaphysis. Magnetic resonance imaging showed ACL tear, complex tear of the posterior horn and body of the medial meniscus, and an absent patella (Figure 3). Computed tomography scans of the knee showed postsurgical changes of ACL reconstruction. The femoral tunnel measured up to 9 × 8 mm and the tibial tunnel measured up to 11 × 6 mm (Figures 4 and 5).

Given the symptoms, physical examination, and imaging findings, the patient was offered surgical treatment of arthroscopic partial medial meniscectomy and revision ACL reconstruction.

In the operating room, general anesthesia was administered, and the patient was placed supine on the operating room table. Under anesthesia, examination of the left knee revealed range of motion of –5° to 145°, grade 2B Lachman, grade 2 pivot shift, and normal varus and valgus stress testing. The right knee examination revealed range of motion of –5° to 145°, negative Lachman, negative pivot shift, and normal varus and valgus stress testing.

Systematic arthroscopic examination of the joint revealed an absent patella and a complex tear of the medial meniscus posterior horn. Fifteen percent of the meniscus over a 10-mm area was debrided with an arthroscopic shaver and biter. The ACL was probed and found to be incompetent with a positive empty-wall sign. The posterior cruciate ligament (PCL) was stable.

Prior ACL sutures were removed with associated scar tissue, and lysis of adhesions was performed. The intercondylar notch was prepared with minimal notchplasty. The femoral tunnel guide pin was established in the medial portal at the anatomic insertion site of the anteromedial and posterolateral bundle junction at the 10 o’clock position and 120° of knee flexion. A guide pin was placed and then reamed to 25 mm. Care was taken to avoid breach of the lateral cortex. The tibial ACL guide at 55° followed by tibial guide pin was placed. Tibial tunnel was core drilled to 9.0 mm and then dilated to 9.5 mm and 10.0 mm, as per the size of the graft.

The Achilles allograft was 110 mm in total length with a 20-mm bone block × 10 mm in diameter. Following graft passage, an 8-mm BioComposite Delta Interference screw (Arthrex, Inc) was placed in the femoral tunnel with solid fixation that was stable to resistance distally. The surgeon ensured that the graft was posterior to the screw in the femoral tunnel. The 10-mm screw did not provide sufficient bone purchase in the tibia, so an 11-mm screw was used. An 11 × 30-mm BioComposite Delta Interference screw was placed in the tibia under >15 pounds of tension after notching the tibial tunnel to give solid fixation to the ACL graft. Because of the risk of screw prominence, 15 mm of the proximal end of the screw was sawed off. The knee could be extended to 0° without any anterior impingement; confirmation of the knee being able to be fully extended without impingement was done prior to fixation distally. The knee was stable with grade 1A Lachman. A 4.75 × 19.1-mm BioComposite SwiveLock anchor (Arthrex, Inc) was placed distal to the tibial tunnel, incorporating sutures of the graft to supplement tibial fixation distally.

**Figure 6.**
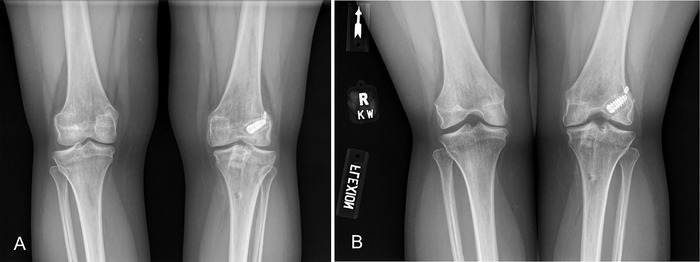
Six-week postoperative anteroposterior radiographs of both knees (A) in standing view and (B) at 45° flexion show appropriate positioning of the tunnels and hardware.

**Figure 7.**
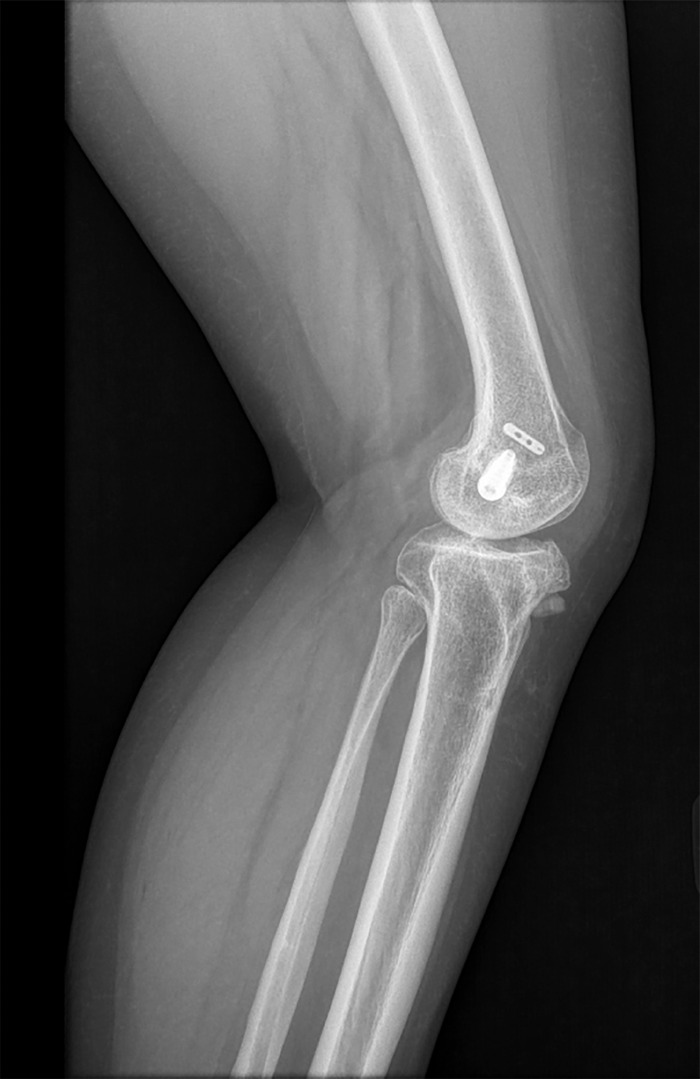
Six-week postoperative lateral radiograph of the left knee shows appropriate positioning of the tunnels and hardware.

The surgical incisions were closed in layers and appropriate dressings applied, along with a brace locked in extension. We followed the arthroscopic ACL reconstruction with allograft rehabilitation guidelines.^14^ The patient remained toe-touch weight-bearing for 4 weeks and mobilized in the brace for 6 weeks. Postoperative radiographs of the knee at 6 weeks showed appropriate positioning of the tunnels and hardware (Figures 6 and 7).

During the next 12 months, the patient progressed well with physical therapy. She demonstrated good pain relief and improvements in range of motion. We used the Single Assessment Numeric Evaluation (SANE) score to collect patient-reported outcome measures. The SANE score, which reflects the patient's reported knee function on a scale of 0 to 100, increased from 30 preoperatively, to 80 at 6 months postoperatively, to 90 at 1 year, and to 100 at 2 years postoperatively. The patient's range of motion at 2-year follow-up was –5° to 145° of flexion. At her 1-year postoperative follow-up, the patient reported that her postoperative outcome surpassed her expectations in terms of pain relief, improved stability, and resumption of routine activity.

## DISCUSSION

Meier-Gorlin syndrome is associated with patellar hypoplasia or aplasia, hypoplastic lateral femur condyle, genu valgum, flattened epiphysis, and joint laxity.^15^ Loeys et al reported that 70% of patients with Meier-Gorlin syndrome have joint abnormalities.^16^

Dudkiewicz and Tanzer reported the case of a 33-year-old patient with Meier-Gorlin syndrome who underwent total knee arthroplasty (TKA) for severe osteoarthritis.^17^ Intraoperatively, the authors found a flattened lateral femur condyle, absent ACL, and thin PCL. They performed a PCL-retaining TKA. At 3-year follow-up, the patient was pain free and was able to perform all activities of daily living.^17^ In 1992, Gorlin provided an update on a 16-year-old patient with Meier-Gorlin syndrome (originally reported in 1975) who had Blount osteochondritis dissecans, patellar aplasia, and osteonecrosis of the lateral femoral condyles. The patient underwent “surgical procedures” on both his knees at age 25 years, but by age 36 years, his knee pain precluded strenuous physical activity.^18^

To our knowledge, literature on revision ACL reconstruction specifically in the absence of the patella is limited, with most data focusing on primary ACL reconstruction following total patellectomy. Revision ACL reconstruction in the absence of the patella presents a surgical challenge because of the altered knee biomechanics, loss of extensor mechanism integrity, and limited graft options. The patella serves as a crucial component in the extensor mechanism and provides an anchor point for commonly used autografts such as the bone-patellar tendon-bone graft.^19^ When the patella is absent, the reconstruction technique must be carefully planned to compensate for the altered kinematics and graft limitations. Our case illustrates the successful use of an allograft with secure femoral and tibial fixation, tailored to a knee without a patella.

We used the SANE score to measure the functional outcome following our patient's surgery. The SANE score is a commonly used outcome measure that has been shown to reliably reflect knee symptoms in ACL reconstruction and to strongly correlate with the Cincinnati Knee Rating System, Lysholm knee score, and the International Knee Documentation Committee subjective knee survey.^20,21^

In our literature review, we could not find a single case of ACL reconstruction in a patient with Meier-Gorlin syndrome. Our experience adds to the growing body of evidence suggesting that with careful planning and graft selection, functional outcomes can be favorable. Close attention to rehabilitation, especially quadriceps strengthening and proprioceptive training, is critical for optimizing results.

## CONCLUSION

To our knowledge, our case is the first report of revision ACL reconstruction in a twin patient with Meier-Gorlin syndrome. The patient achieved excellent outcomes. Her SANE score improved from 30 to 90 at 1 year and increased to 100 at 2 years postoperatively. In addition, she reported full pain relief and restored daily function, demonstrating that ACL reconstruction is feasible in patients with an absent patella.

## Notes

*This article meets the Accreditation Council for Graduate Medical Education and the American Board of Medical Specialties Maintenance of Certification competencies for Patient Care and Medical Knowledge.*
